# Conditional Expression of Human PPAR**δ** and a Dominant Negative Variant of hPPAR**δ* In Vivo*

**DOI:** 10.1155/2012/216817

**Published:** 2012-03-21

**Authors:** Larry G. Higgins, Wojciech G. Garbacz, Mattias C. U. Gustafsson, Sitheswaran Nainamalai, Peter R. Ashby, C. Roland Wolf, Colin N. A. Palmer

**Affiliations:** ^1^CRUK Molecular Pharmacology Unit, Medical Research Institute, University of Dundee, Ninewells Hospital and Medical School, Dundee DD1 9SY, UK; ^2^Department of Laboratory Medicine, Division of Medical Microbiology, Lund University, Sölvegatan 23, SE-223 62 Lund, Sweden; ^3^Division of Molecular Medicine, Medical Science Institute, College of Life Sciences, University of Dundee, Dundee DD1 4HN, UK

## Abstract

The nuclear receptor, NR1C2 or peroxisome proliferator-activated receptor (PPAR)-**δ**, is ubiquitously expressed and important for placental development, fatty acid metabolism, wound healing, inflammation, and tumour development. PPAR**δ** has been hypothesized to function as both a ligand activated transcription factor and a repressor of transcription in the absence of agonist. In this paper, treatment of mice conditionally expressing human PPAR**δ** with GW501516 resulted in a marked loss in body weight that was not evident in nontransgenic animals or animals expressing a dominant negative derivative of PPAR**δ**. Expression of either functional or dominant negative hPPAR**δ** blocked bezafibrate-induced PPAR**α**-dependent hepatomegaly and blocked the effect of bezafibrate on the transcription of PPAR**α** target genes. These data demonstrate, for the first time, that PPAR**δ** could inhibit the activation of PPAR**α* in vivo* and provide novel models for the investigation of the role of PPAR**δ** in pathophysiology.

## 1. Introduction

There are three PPAR isoforms, PPAR*α*, *β*/*δ* (herein referred to as *δ*), and *γ*. PPAR*α* is found in tissues with a high rate of fatty acid catabolism where when agonist bound it activates the expression of genes involved in peroxisomal *β*-oxidation and mitochondrial *β*- and *ω*-oxidation and plays an important role in systemic fat catabolism, in the generation of ketone bodies and stimulating gluconeogenesis [1, 2]. Fibrate drugs cause PPAR*α*-dependent liver peroxisome proliferation, hepatomegaly, and subsequently hepatocarcinoma when fed to rodents [2, 3]. Fibrate drugs are used successfully in the clinic to treat hyperlipidaemia and do not cause liver problems, most probably due to the lower level of PPAR*α* in human liver [4, 5].

There are two forms of PPAR*γ*, (*γ*1 and *γ*2, with differing amino termini). PPAR*γ* is important for fatty acid and triglyceride anabolism and storage and essential for the differentiation of adipocytes [2]. 

PPAR*δ* is the least known isoform in terms of biological function and the most abundant PPAR isoform in all tissues except liver and adipose tissue in rodents. PPAR*δ* is downregulated in liver and kidney in response to fasting [6]. In all tissues examined, PPAR*δ* protein was predominantly localised in the nucleus and could be immunoprecipitated with RXR*α* [7]. *ppar*δ*^−/−^* mice have impaired placental function leading to viability problems [8–10]. Surviving knockouts were leaner and had reduced adipose stores, but this does not appear to be a direct effect of PPAR*δ*, since adipose-specific *ppar*δ*^−/−^* mice did not show the same phenotype [8]. PPAR*δ* knockouts also showed increased keratinocyte proliferation in response to topical application of tetradecanoylphorbol acetate [9, 10].

PPAR*δ* has been shown to play an important role in cellular differentiation [11–13] and in regulating energy homeostasis [8, 14]. Mice generated with PPAR*δ* constitutively active in adipocytes were found to be lean, and leptin receptor mutant mice showed improved metabolic activity and reduced fat deposits when fed GW501516 (a PPAR*δ*-specific agonist) [15]. Muscle-specific PPAR*δ* overexpression resulted in a net increase of muscle fibres with an oxidative metabolic phenotype with a concomitant decrease in body fat [16]. Comparably, mice with a cardio-restricted deletion of *PPAR*δ** showed decreased basal myocardial fatty acid oxidation followed by chronic lipid accumulation in the heart that led to cardiac hypertrophy and congestive heart failure [17].

The involvement of PPAR*δ* in cancer promotion in various organs is clear; however, there is controversy about whether ligand activation of PPAR*δ* is pro- or anti-tumourigenic. Following initiation, increased breast tumour development was evident in animals fed with GW501516 [18]. In animal models of colorectal cancer, PPAR*δ* has been shown to both inhibit and promote the growth of intestinal polyps [8, 19, 20].

One study has shown that PPAR*δ* was able to attract transcriptional corepressors and when bound to DNA in the absence of a PPAR*δ* agonist more effectively than PPAR*α* or PPAR*γ* [21]. On the other hand, treatment of wild-type adipocytes with troglitazone, a potent PPAR*γ* ligand, caused upregulation in PPAR*γ* activity as measured by adipocyte differentiation and lipid accumulation assays. This effect was also almost entirely preserved in PPAR*δ*-null adipocytes [22]; however, in another study, authors conclude that PPAR*δ* suppressed PPAR*γ* activity, but downregulation of PPAR*δ* expression did not increase PPAR*γ* expression levels [23]. A different study has shown that PPAR*δ* is able to repress both PPAR*α* and *γ*-dependent gene expression *in vitro *[24]. Since PPAR*δ* is the predominant PPAR isoform in many tissues [6], it was proposed that PPAR*δ* could act as a PPRE gateway receptor [24]. However, deletion of the carboxyterminal exon of PPAR*δ* did not result in increased expression of PPAR*α* target genes in liver [25].

With confusing and opposing results being observed using knockout and drug models, we decided to take an alternative route to provide genetic models for both gain and loss of function of PPAR*δ*. We show that we can control expression of hPPAR*δ* and hPPAR*δ*ΔAF2 (a dominant negative derivative lacking the 11 carboxy-terminal aminoacid residues [26, 27]) in transgenic animals and that we can modulate PPAR-dependent gene expression in liver as well as the hepatomegaly associated with activation of PPAR*α*. These new mouse models for studying PPAR*δ* biology should thus be useful in resolving some of the uncertainties regarding this receptor. 

## 2. Results

### 2.1. Conditional Expression of hPPAR*δ* and hPPAR*δ*ΔAF2 in Transgenic Mice

**Figure 1:**
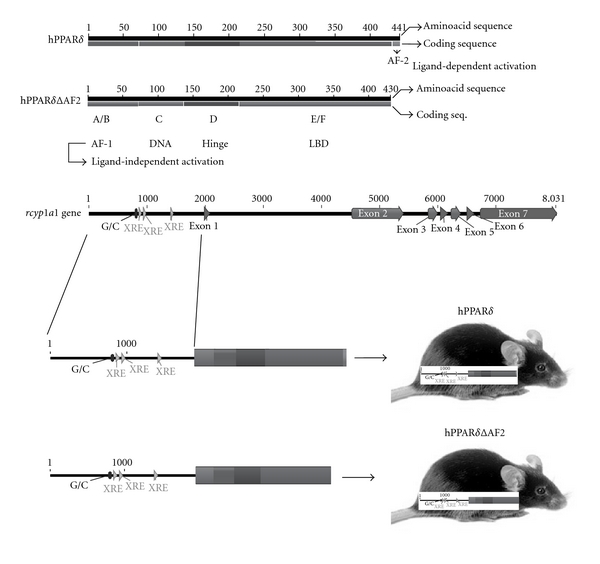
Graphic depicting the generation of mice conditionally expressing hPPAR*δ* and hPPAR*δ*ΔAF2 transgenes. Mice harbouring transgenes, encoding the coding sequence of hPPAR*δ* or hPPAR*δ*ΔAF2 fused to the rat *Cyp1a1* gene promoter and offering tight control of XRE enhancer driven transgene expression.

Two mouse lines were generated that conditionally express human PPAR*δ* and a dominant negative derivative thereof, hPPAR*δ*ΔAF2 (Figure 1). This approach allows for the manipulation of the levels of PPRE signaling through the conditional production of human PPAR*δ* and a dominant negative derivative, hPPAR*δ*AF2, in transgenic mice. The *Cyp1a1* gene is tightly regulated *in vivo *and its expression is wholly dependent on the arylhydrocarbon receptor (AhR), functioning as a heterodimer with the AhR nuclear translocator, Arnt, that binds the xenobiotic response element enhancer sequence in the regulatory 5′ UTR of the *Cyp1a1* gene. The promoter of the rat *Cyp1a1* gene has been used to conditionally express several genes in transgenic models [28–30]. The expression of genes under the control of the *Cyp1a1* promoter is achieved by the administering of compounds that activate XRE-driven gene expression.

**Figure 2:**
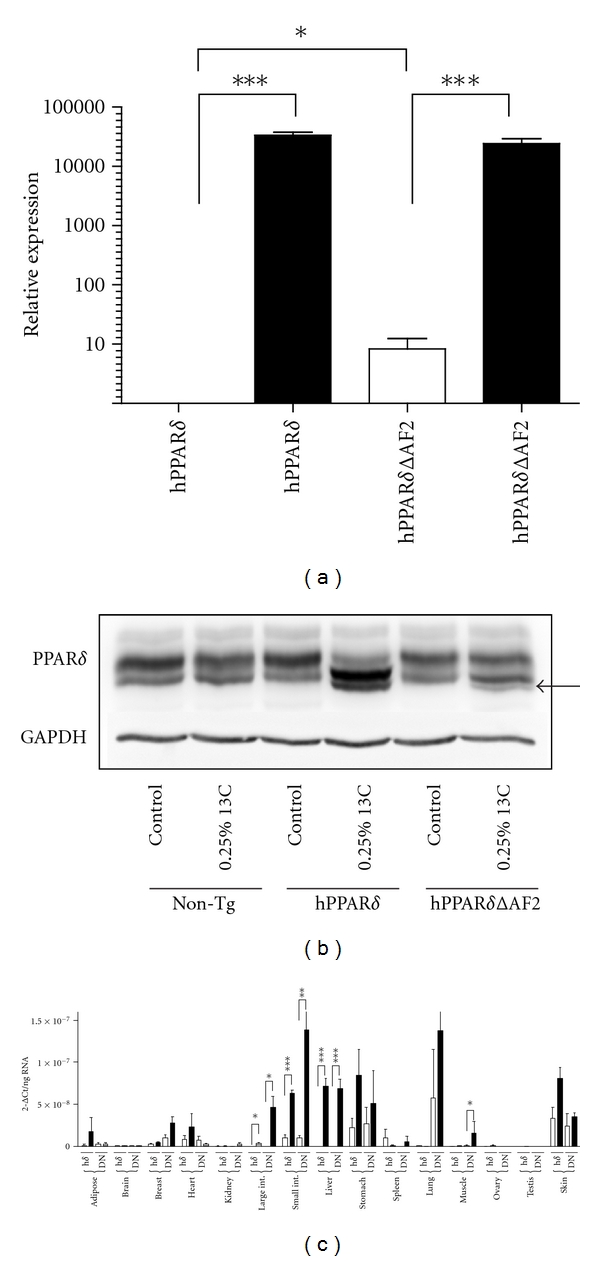
Mice conditionally express hPPAR*δ* or hPPAR*δ*ΔAF2 transgenes in response to feeding I3C. Animals (*n* = 3 per group, 10 weeks old, mixed male and female) were placed either on control diet or a diet containing I3C (0.25% (w/w)) for 5 days. (a) hPPAR*δ* and hPPAR*δ*ΔAF2 exhibit low basal mRNA expression (open bars) and highly inducible I3C-dependent expression (black bars) in mouse liver. (b) hPPAR*δ* and hPPAR*δ*ΔAF2 protein is expressed in livers in response to dietary supplementation with I3C. (c) hPPAR*δ* (h*δ*) and hPPAR*δ*ΔAF2 (DN) mRNA expression in various mouse organs in response to normal diet (open bars) and I3C-supplemented diet (black bars). Statistical significance where indicated was analysed using Mann-Whitney nonparametric test in GraphPad Prism version 5.0c, (GraphPad Software, San Diego, CA, USA). Significance is portrayed as (*P ≤ 0.05; **P ≤ 0.01; ***P ≤ 0.001).

I3C, found in cruciferous vegetables, is converted to polyaromatic indolic compounds in the acid environment of the stomach and produces a potent and dose-dependent activation of XRE driven gene expression [31]. Feeding transgenic mice with a diet supplemented with I3C (0.5% (w/w)) or (0.25% (w/w)) resulted in a similar induction of both hPPAR*δ* and hPPAR*δ*ΔAF2 transgenes in these animals (data not shown). Based on this result, we fed nontransgenic, hPPAR*δ* and hPPAR*δ*ΔAF2 transgenic mice either on control diet or a diet supplemented with I3C (0.25% (w/w)) for 5 days and examined transgene expression in a range of organs (Figure 2). hPPAR*δ* mRNA from livers of mice fed control diet was virtually undetectable, but hPPAR*δ* message increased approximately 30,000-fold upon feeding I3C (0.25% (w/w)) for 5 days (Figure 2(a)). By contrast, hPPAR*δ*ΔAF2 animals on control diet had approximately 10-fold greater basal expression of the transgene than did the hPPAR*δ* mice on the same diet. However, when these animals were fed on a diet containing I3C, the hPPAR*δ*ΔAF2 transgene expression in the liver increased (2900-fold) to levels similar to that of animals expressing the hPPAR*δ* transgene (Figure 2(a)). Although I3C induced mRNA levels of both the hPPAR*δ* and hPPAR*δ*ΔAF2 transgenes to similar levels in these mice, the corresponding protein expression in the livers of these mice differed. Using a monoclonal antibody raised against hPPAR*δ*, animals expressing the hPPAR*δ* transgene showed a robust increase in hPPAR*δ* protein in their livers when fed on an I3C-supplemented diet compared to those on control diet (Figure 2(b)). When liver hPPAR*δ*ΔAF2 protein was examined in mice expressing this transgene, there was found to be a much less robust increase in protein expression (Figure 2(b), see arrow). It is possible that hPPAR*δ*ΔAF2-truncated protein may be less stable in the livers of these animals than the human full length form. 

Low basal expression of both hPPAR*δ* and hPPAR*δ*ΔAF2 mRNA was also observed in the adipose, brain, kidney, large intestine, muscle, ovary, and testis of animals on control diet with no significant induction of message by I3C observed in the brain, adipose, breast, heart, kidney, stomach, spleen, lung, muscle, ovary, or testis of mice harbouring either transgene. The large intestine showed the largest induction of both transgenes in response to I3C treatment. A greater basal expression of hPPAR*δ* transcript was seen in breast, heart, small intestine, and stomach. These organs showed an increase in hPPAR*δ* expression upon feeding animals with an I3C-supplemented diet, but this increase was significant only in small intestine, *P* = 0.0007 and *P* = 0.005 for hPPAR*δ* and hPPAR*δ*ΔAF2, respectively. Lung and muscle showed a very different pattern of expression from the other tissues in that basal expression of hPPAR*δ* was low and not inducible by I3C dietary supplementation. In lung, the basal expression of hPPAR*δ*ΔAF2 was high and induction by I3C observed but not significant. In muscle, however, basal expression of hPPAR*δ*ΔAF2 was low but induced by I3C in the diet, *P* = 0.033. hPPAR*δ* is known to be expressed in the sebaceous gland of the skin, and is induced upon feeding I3C in keratinocytes of hPPAR*δ* transgenic mice [32].

### 2.2. Induction and Activation of hPPAR*δ* Causes Body Weight Loss

**Figure 3:**
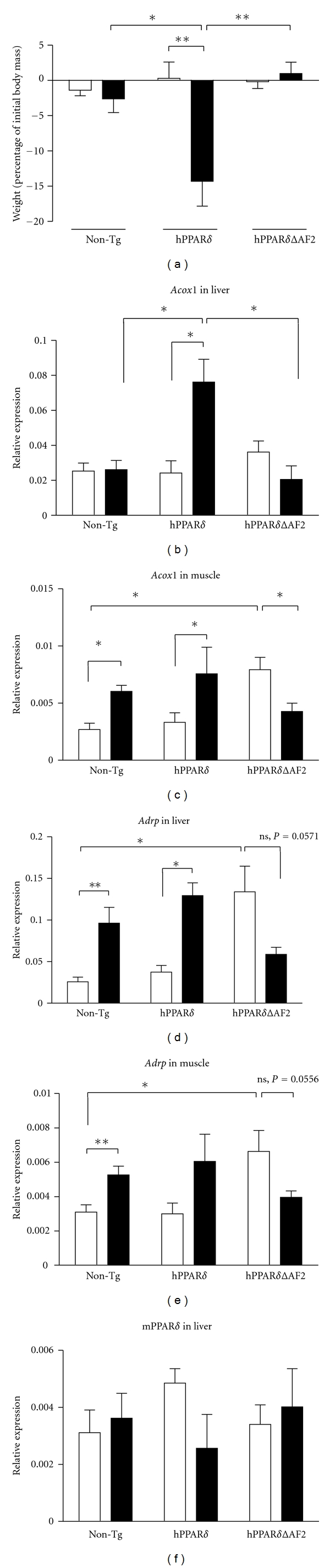
Mice expressing functional hPPAR*δ* and hPPAR*δ*ΔAF2 protein. Animals (*n* = 5 per group, 12 weeks old, mixed males and females) were placed on a diet containing I3C (0.25% (w/w)) (open bars) or a diet containing I3C (0.25% (w/w)) and GW501516 (0.0025% (w/w)) (black bars) for 14 days. (a) body weight of nontransgenic (Non-Tg), hPPAR*δ* and hPPAR*δ*ΔAF2 transgenic mice expressed as a percentage of initial body weight. (b–f) mRNA expression of *Acox1* in liver (b), muscle (c), mRNA expression of *Adrp* in liver (d), muscle (e), and mouse endogenous PPAR*δ* (f) following treatments outlined above. Statistical significance (*P ≤ 0.05; **P ≤ 0.01; ***P ≤ 0.001) where indicated was analysed using Mann-Whitney non-parametric test in GraphPad Prism version 5.0c, (GraphPad Software, San Diego, CA, USA).

PPAR*δ* activation is known to produce favorable metabolic effects that include weight loss with increased metabolic rate in skeletal muscle, improved exercise endurance and insulin sensitivity [15, 33]. To investigate the role of activating hPPAR*δ* and hPPAR*δ*ΔAF2 on body weight, mice were fed either on normal diet supplemented with I3C (0.25% (w/w)) (I3C diet) or on an I3C diet supplemented with GW501516 (0.005% (w/w)) for 14 days. There was no difference in the weights of either nontransgenic, hPPAR*δ* or hPPAR*δ*ΔAF2 transgenic mice fed on I3C diet (Table 1 and Figure 3(a)). In nontransgenic mice, there was a small although significant reduction in body weight when mice were given I3C diet containing GW501516 likely as a response of endogenous PPAR*δ* activation by the ligand (Table 1 and Figure 3(a)). In animals carrying hPPAR*δ* placed on the GW501516 diet, there was a marked reduction in body weight (direct weight difference (*P* = 0.017) and percentage of body weight (*P* = 0.008)) over the two-week period that was completely absent in nontransgenic animals or animals expressing hPPAR*δ*ΔAF2 (Table 1 and Figure 3(a)).

### 2.3. GW501516-Dependent Activation of Gene Expression Is Abolished, or Reversed, by hPPAR*δ*ΔAF2

hPPAR*δ*ΔAF2 has been shown to act in a dominant negative fashion *in vitro*. This effect was augmented by the addition of a PPAR*δ* agonist [26]. To examine the effect of conditionally expressing hPPAR*δ*ΔAF2 on PPAR*δ* target gene expression, animals were fed on a diet containing either I3C (0.25% (w/w)) only or on a diet containing I3C and GW501516 (0.25% (w/w) and 0.0025% (w/w), resp.) for 2 weeks prior to liver and muscle tissue being harvested and analyzed for relative mRNA expression levels of *Acox1*, *Adrp*, and mouse PPAR*δ* (Figures 3(b)–3(f)). 

*Acox1*, a known PPAR*δ* target encoding the first enzyme of the fatty acid *β*-oxidation pathway, was upregulated in muscle (*P* = 0.016) but not in liver of nontransgenic animals fed a diet containing GW501516 (Figures 3(b) and 3(c)). This muscle upregulation of *Acox1 *is most likely attributable to activation of endogenous mPPAR*δ*. We found, however, that the hPPAR*δ* transgene is required for up-regulation of *Acox1 *mRNA in liver (*P* = 0.036), indicating the presence of a functional hPPAR*δ* protein. Up-regulation of muscle *Acox 1* in response to GW501516 was also observed in hPPAR*δ* transgenic animals (*P* = 0.015) (Figure 3(c)). hPPAR*δ*, however, is not likely to be involved in this response as expression of this transgene was not seen in muscle of hPPAR**δ**-transgenic mice when administered I3C in the diet (Figure 2(c)), and a similar level of induction of *Acox1* was seen in nontransgenic mice (Figure 3(c)). In contrast, hPPAR*δ*ΔAF2 was shown to be inducible in both liver and muscle of hPPAR*δ*ΔAF2 transgenic mice by I3C (Figure 2(c)). The GW501516-dependent up-regulation of *Acox1* in both liver and muscle was completely inhibited in the hPPAR*δ*ΔAF2 transgenic mice (Figures 3(b) and 3(c)). Interestingly, *Acox1* expression was significantly increased in mice expressing hPPAR*δ*ΔAF2 compared to nontransgenic mice in the absence of the PPAR*δ* agonist. The increase in *Acox1* expression was lowered in the presence of PPAR*δ* agonist (Figure 3(c)), indicating agonist-induced repression by hPPAR*δ*ΔAF2 as described by Gustafsson et al. [26].

*Adrp* another PPAR target gene encodes a protein that coats lipid droplets and is expressed during lipid accumulation. Treatment of mice with GW501516 for 2 weeks leads to up-regulation of *Adrp* mRNA in both liver and muscle of nontransgenic mice (*P* = 0.008 for both organs). Again this is most likely due to GW501516 activation of endogenous mouse PPAR*δ* (Figures 3(d) and 3(e)). In transgenic mice expressing hPPAR*δ*, there was an up-regulation of *Adrp *by GW501516 in liver (*P* = 0.036) resulting in a 3.4-fold activation. Nontransgenic animals displayed an identical (3.4-fold) activation of *Adrp by GW501516* in their livers (Figure 3(d)). Levels of *Adrp* were also increased by GW501516 in the muscle of these animals but did not reach significance (*P* = 0.151) (Figure 3(e)). Thus, the extent of basal and GW501516-induced expression of *Adrp* in liver and muscle do not seem to be affected by hPPAR*δ* transgene. Interestingly, basal levels of *Adrp* were significantly higher in mice expressing hPPAR*δ*ΔAF2 compared to either nontransgenic or hPPAR*δ* transgenic counterparts on I3C diet (*P* = 0.016 for both organs) (Figures 3(d) and 3(e)). The ability of hPPAR*δ*ΔAF2 to repress expression of *Adrp* mRNA in the presence of GW501516 was evident in both liver and muscle of these animals with *Adrp* mRNA levels approaching the basal levels in nontransgenic and hPPAR*δ* transgenic animals on I3C diet (Figure 3). This is most likely the result of ligand-augmented repression by hPPAR*δ*ΔAF2 described by us previously [26]. Endogenous mouse PPAR*δ* mRNA levels showed no change in expression pattern between treatment or transgene groups (Figure 3(f)).

Treatment of hPPAR*δ* mice with GW501516 led to a greater than 200% increase in the accumulation of hepatic triglycerides when compared to untreated hPPAR*δ* animal fed on control diet (*P* = 0.0024; Table 1).

Surprisingly, hPPAR*δ*ΔAF2 animals fed on control diet had liver triglycerides levels comparable to the hPPAR*δ* mice fed the ligand-supplemented diet. However, hPPAR*δ*ΔAF2 animals treated with GW501516 had a lower level of hepatic triglycerides (−108%), when compared to hPPAR*δ*ΔAF2 fed on control diet (*P* = 0.0023; Table 1). Nontransgenic animals fed a diet supplemented with GW501516 displayed an increased hepatic triglyceride levels (122%) when compared to nontransgenic animals on control diet (*P* = 0.0011; Table 1).

### 2.4. Expression of PPAR*δ* and PPAR*δ*ΔAF2 Modulate Bezafibrate-Induced Hepatomegaly

**Figure 4:**
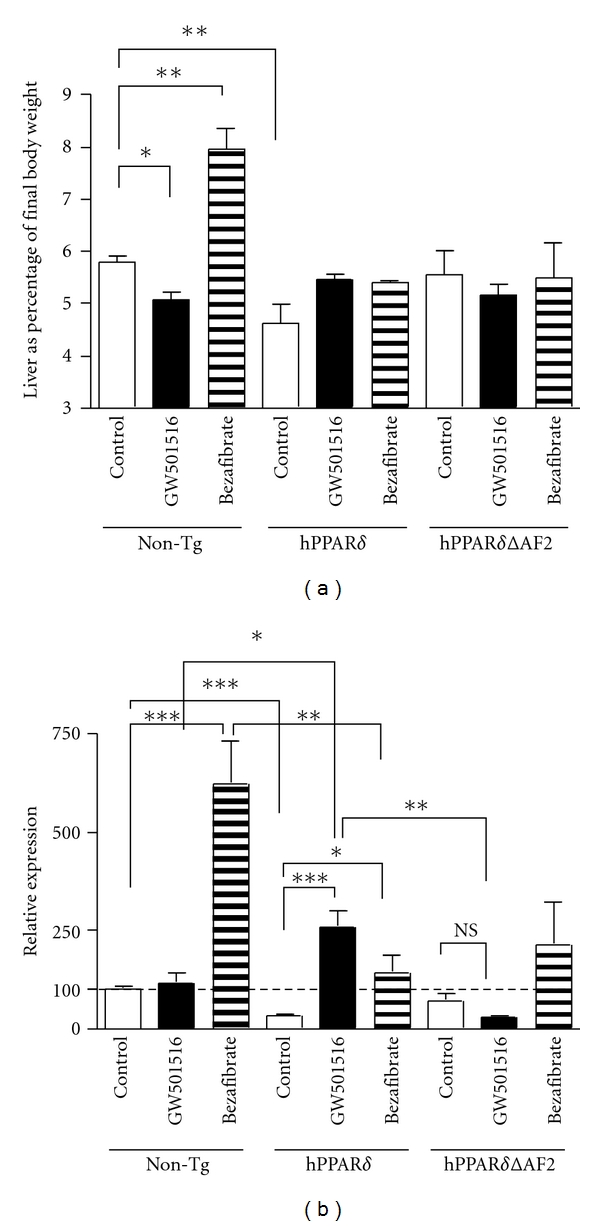
Expression of hPPAR*δ* and hPPAR*δ*ΔAF2 abolish bezafibrate-induced hepatomegaly in liver. Animals (*n* = 2–4 animals per group) were fed a diet containing I3C (0.5% (w/w)) for 10 days prior to being placed on diets containing I3C (0.5% (w/w)) (Control diet; open bars), or a diet containing I3C (0.5% (w/w)) and GW501516 (0.005% (w/w)) (GW501516 diet; black bars) or a diet containing I3C (0.5% (w/w)) and bezafibrate (0.5% (w/w)) (bezafibrate diet; striped bars) for a further 10 days. (a) Liver weights of animals are expressed as a percentage of body weight. (b) Liver *Acox1* mRNA expression. Graphs were analysed using the Mann-Whitney non-parametric test in GraphPad Prism version 5.0c, GraphPad Software, San Diego, California, USA. Significance is indicated as (*P ≤ 0.05; **P ≤ 0.01; ***P ≤ 0.001).

Bezafibrate has been shown to act as a dual PPAR*α*/PPAR*δ* agonist in mice and to cause hepatomegaly when administered in the diet at 0.5% (w/w). This response is completely absent in PPAR*α* knockout mice [25]. To investigate whether or not PPAR*δ* or PPAR*δ*ΔAF2 expression could modulate PPAR*α*-dependent hepatomegaly in mice fed with bezafibrate, mice were placed on a diet containing I3C (0.5% (w/w)) for 10 days to induce PPAR*δ* transgene expression. Following this, GW501516 (0.005% (w/w)), a PPAR*δ*-specific ligand, or the PPAR*α*/*δ* dual ligand, bezafibrate (0.5% (w/w), was introduced to the diet containing I3C (0.5% (w/w)) for a further 10 days. Subsequently animals were sacrificed and liver weights recorded. Nontransgenic mice showed a decrease in liver weight (*P* = 0.032) when fed on GW501516-supplemented diet (Figure 4(a)). In contrast, nontransgenic animals fed bezafibrate supplemented diet displayed an increase in liver weight (*P* = 0.003) (Figure 4(a)). This finding is in agreement with previously published data [25]. Animals expressing hPPAR*δ* or hPPAR*δ*ΔAF2 showed no change in liver weight when fed with either bezafibrate or GW501516 containing diets (Figure 4(a)).

### 2.5. PPAR*δ* and PPAR*δ*ΔAF2 Modulate Bezafibrate Regulation of Gene Expression in the Liver

To investigate whether or not the ability of hPPAR*δ* and hPPAR*δ*ΔAF2 to alleviate bezafibrate-induced hepatomegaly in these mice was associated with a repression of bezafibrate-induced gene expression by the hPPAR*δ* and hPPAR*δ*ΔAF2 transgenes, we examined mRNA expression from the livers of mice treated with bezafibrate or GW501516. The level of *Acox1* in nontransgenic mouse liver was strongly induced by bezafibrate but not by GW501516 (Figure 4(b)). In contrast, hPPAR*δ* transgenic mice displayed decreased constitutive levels of *Acox1* (*P* = 0.003), but retained inducibility with both GW501516 and bezafibrate (*P* = 0.001 and *P* = 0.011, resp.). Taken together, these observations suggest, that hPPAR*δ* represses basal expression of *Acox1*, and that GW501516 activated-hPPAR*δ* induces expression of *Acox1* in the livers of these animals (Figure 4(b)). The level of *Acox1* expression achieved by bezafibrate in the hPPAR*δ* transgenic mice was much lower than that achieved in the nontransgenic animals (*P* = 0.0095). There were no significant changes in the liver expression levels of *Acox1* in the hPPAR*δ*ΔAF2 animals in response to either GW501516 or bezafibrate (Figure 4(b)). Of note, even though the change was not statistically significant, we observed a trend to a ligand-dependent repression of PPAR*δ* target gene expression in hPPAR*δ*ΔAF2 animals similar to that observed in *in vitro* cellular assays [26] and in (Figures 3(b)–3(e) and Figure 4(b)). These observations provide further evidence for the dominant negative role of the hPPAR*δ*ΔAF2 transgene in regulating both GW501516 (PPAR*δ* specific agonist) and bezafibrate (PPAR*α*/*δ* dual agonist) gene induction *in vivo* (Figure 4(b)).

## 3. Discussion

Attempts to resolve PPAR*δ* biology have proven difficult with several groups reporting contradictory results obtained from both genetic and pharmaceutical modulation of PPAR*δ* activity [8, 19, 20]. We believe that the interpretation of PPAR*δ* experimentation results is confounded by the ability of PPAR*δ* to function as a repressor and activator of transcription depending on ligand binding status. We hypothesised that a more subtle modification of PPAR*δ* *in vivo* would provide a useful tool to help delineate the biology of this nuclear receptor. To that end, we have in this study described two transgenic mouse models that conditionally expresses either human PPAR*δ* or a dominant negative derivative of hPPAR*δ* lacking the carboxy terminal 11 aminoacids comprising the activation function 2 domain of the protein. Both transgenes are functional, and human *PPAR*δ** mRNA is detectable in a range of tissues and protein expression is evident in the liver (Figure 2). In addition, these transgenic animals are refractory to bezafibrate-induced hepatomegaly (Figure 4) indicating that these mice are synthesising functional human PPAR*δ* protein *in vivo*. In other model of humanized mice generated by Gross et al., the endogenous murine PPAR*δ* was replaced with human PPAR*δ*. Gene expression profiling in liver, soleus muscle, and macrophages showed similar gene patterns regulated by mouse and human PPAR*δ*. In terms of regulation of lipid metabolism and inflammation, authors indicate that human PPAR*δ* can compensate for mouse PPAR*δ* [34].

The regulation of the rat *Cyp1a1 *gene is well characterized and tightly controlled, and its promoter has been used for the conditional expression of genes in transgenic animals [28–30]. Our results demonstrate that expression of hPPAR*δ* and hPPAR*δ*ΔAF2 is highly inducible in the liver and intestine, but not in all tissues examined. For example, the stomach displayed a high basal expression of both PPAR*δ* transgenes that was not significantly induced by feeding I3C. In skin there was no significant induction of mRNA for either transgene. However, administration of dietary GW501516 to hPPAR*δ* mice results in skin thickening with psoriasis-like lesions. In the skin of hPPAR*δ* mice, basal hPPAR*δ* protein is detected in the sebaceous gland. When exposed to I3C, these animals show inducible *PPAR*δ** mRNA expression in keratinocytes [32].

When the liver was examined for hPPAR*δ* and hPPAR*δ*ΔAF2 protein expression, mRNA and protein levels did not correlate well. In hPPAR*δ* transgenic animals, there was a clear hPPAR*δ* protein signal that was not substantially elevated above endogenous mouse PPAR*δ*. In contrast, hPPAR*δ*ΔAF2 protein expression was very weak despite a robust I3C-dependent increase in mRNA expression (Figure 2). Notwithstanding this, the hPPAR*δ*ΔAF2 protein was clearly functional. In animals expressing hPPAR*δ*ΔAF2, there was a complete inhibition of PPAR*δ*-mediated induction of *Acox1* or *Adrp* in the liver and muscle in the presence of the PPAR*δ* agonist GW501516 (Figures 3(c), 3(d), and 3(e)).

One noticeable observation is the higher basal levels of both *Acox1* in muscle and *Adrp* in liver and muscle of animals expressing hPPAR*δ*ΔAF2 protein without an added PPAR*δ* agonist in the diet. This may be as a result of the nonliganded dominant negative protein relieving endogenous mouse PPAR*δ*-dependent repression of these genes as suggested by previous studies, particularly by the sequestration of RXR species from PPAR*δ*-RXR heterodimers leading to PPRE being occupied by DR1 binding positive regulators that do not require RXR such as HNF4 or COUP-TFII [35–37]. Further work is required to confirm this at the genomic level.

In contrast to nontransgenic mice, the levels of *Acox1* and *Adrp* were actually reduced in the presence of PPAR*δ* agonist in the hPPAR*δ*ΔAF2 mice when compared to the levels in animals not receiving GW501516. This is in line with our previous observations where *in vitro* transfection experiments suggested that the hPPAR*δ*ΔAF2-mediated repression is enhanced by a PPAR*δ* agonist (most probably due to agonist enhanced hPPAR*δ*ΔAF2/RXR heterodimerisation and thus increased PPRE affinity) [26]. This complex relationship between ligand-activated and dominant negative PPAR*δ* in the control of gene expression reflects the findings of recent genomewide expression and chromatin immunopreciptation studies by Adhikary et al., (2011), where differing modes of target gene regulation by PPAR*δ* have been defined. In these studies, PPAR*δ* was shown to elicit 3 differing transcriptional responses; (a) type 1 response: genes that were up-regulated by siRNA knock down of PPAR*δ*, but were not induced by GW501516, (b) type II response: genes that were up-regulated by knock down of PPAR*δ* and could be up-regulated by GW501516, and (c) type III response: genes that are downregulated by PPAR*δ* siRNA that then showed either no response or a weak induction by GW501516 [38]. Ability to repress the gene expression by PPAR*δ* was also shown in a study carried out by Kino et al.. In this work, overexpression of PPAR*δ* enhanced the suppressive effect of GW501516 on transcriptional regulation of Interleukin-6 [39]. These observations support the complex interaction between activation and expression of PPAR*δ* and the regulation of specific gene targets.

Ligand-activated hPPAR*δ* did not increase mRNA levels of *Acox1 Adrp*  in muscle, or *Adrp* in liver over and above that seen by endogenous ligand-activated mPPAR*δ* (Figure 3). In liver, hPPAR*δ* mRNA and protein was up-regulated by feeding I3C in the diet. In the case of liver, this resulted in a ligand-dependent hPPAR*δ*-specific activation of *Acox1*. *Acox1* was not induced by GW501516 alone in nontransgenic livers, suggesting that endogenous mPPAR*δ* does not affect *Acox1* expression in the liver of these animals but does influence* Acox1* expression in the muscle (Figures 3(b) and 3(c)). *Adrp* expression, on the other hand, is activated by ligand-activated endogenous mouse PPAR*δ* in both liver and muscle, but not modulated by ligand-activated conditionally-expressed human PPAR*δ* in either of these organs. These results may highlight species differences in PPAR*δ* and how it modulates gene expression differently depending on its environment. Taken in context of the work carried out by Adhikary et al. [38], PPAR*δ* elicits a type II transcriptional response in regulating *Acox1* in mouse muscle and *Adrp* in liver and muscle, but mouse PPAR*δ* does not regulate *Acox1* in mouse liver. Interestingly Adhikary et al. note that “the magnitude of induction by ligand approaches the effect of PPAR*δ* depletion for individual genes showing a type II response.” This is mirrored exactly in this model system. The induction (or relief of repression) of muscle *Acox1* and liver and muscle *Adrp* mRNA observed in the presence of hPPAR*δ*ΔAF2 has a magnitude identical to the induction observed in the presence of GW501516 (Figures 3(c), 3(d), and 3(e)). Another striking observation in removing the AF2 domain of PPAR*δ* (PPAR*δ*ΔAF2) results in certain target genes being transcriptionally activated a similar manner to treatment of wild-type mice by the PPAR*δ* ligand GW501516. The opposite is also true, hPPAR*δ*ΔAF2 activated by GW501516 results in gene expression levels identical to those seen in hPPAR*δ* transgenic animals in the absence of GW501516 (Figures 3(c), 3(d), and 3(e)). There is no evidence that hepatic levels of endogenous mouse PPAR*δ* mRNA are being affected by transgene induction or GW501516 treatment (Figure 3(f)). PPAR*δ* is known to be induced by exercise, fasting, and other factors rather than by self-activation [40].

PPAR*δ* has a role in energy homeostasis as a key regulator of fatty acid oxidation, is expressed in skeletal muscle, with a higher expression in soleus muscle. Soleus muscle consists of fatigue resistant type 1 muscle fibres that have a high mitochondria content and use oxidative metabolism for energy production. Activation of PPAR*δ* is known to promote weight loss [15, 33] that was particularly evident when hPPAR*δ* animals were fed a diet containing GW501516 (Figure 3). Interestingly, the increased weight loss seen in the mice expressing hPPAR*δ* cannot be a strictly muscle effect in these mice, as hPPAR*δ* was not induced in muscle of these animals (Figure 2(c)).

So far, role of PPAR*δ* in promoting or preventing hepatic steatosis is an open question [14, 41]. In nontransgenic mice, 2 weeks of PPAR*δ* ligand treatment caused only moderate (2-fold) fluctuations in liver triglycerides (Table 1), whereas in human PPAR*δ* transgenic mice, two weeks of GW501516 treatment was sufficient to cause a significant accumulation of hepatic triglycerides (Table 1). A higher level of triglycerides was also found in livers of hPPAR*δ*ΔAF2 animals on control diet, but not in hPPAR*δ*ΔAF2 mice fed with a GW501516-supplemented diet. This role of the dominant negative enabling a “reverse agonism” action of GW501516 was first seen in our *in vitro* work, and it is intriguing that it is seen so clearly with the hepatic lipid accumulation as well as the target gene expression. This mechanism of this regulation of hepatic triglyceride accumulation is not clear and requires further investigation.

It is not unusual for PPARs to behave differently across species. PPAR*α* is employed as a successful target for pharmaceutical intervention in humans with fibrate drugs being important in treatment of dyslipidemia and cardiovascular disease [42]. In rodents, however, fibrate drugs cause hepatomegaly and eventually hepatic carcinoma [2], which is not seen in humans, and is reflected in a much lower level of expression of PPAR*α* and thus a different balance between PPAR*α* and PPAR*δ* in the human liver [4]. This interaction between PPAR*α* and PPAR*δ* levels in the regulation of hepatomegaly was borne out by our observation that bezafibrate-induced hepatomegaly was blocked by hPPAR*δ*ΔAF2. In addition, expression of hPPAR*δ* blocked bezafibrate-dependent hepatomegaly and modulated bezafibrate-dependent gene expression. Taken together, this data demonstrates that unliganded PPAR*δ* can, in certain circumstances, inhibit the action of PPAR*α*.

In summary, there have been many conflicting and confusing studies attempting to understand PPAR*δ* biology *in vivo*, which are complicated by PPAR*δ* being able to both repress and activate gene expression. Genetic models of deleting *PPAR*δ**  have been very insightful for elucidating the biology of this nuclear receptor. By conditionally expressing hPPAR*δ* and hPPAR*δ*ΔAF2 in an otherwise normal endogenous mouse PPAR*δ* background, we sought to generate animal models that would allow a more subtle examination of PPAR*δ* biology without the complication of removing or inactivating a protein with a dual function. These animals will provide a useful tool to complement studies with knockout and drug models to help resolve the confusing results in the literature regarding PPAR*δ* biology.

## 4. Materials and Methods

### 4.1. Reagents

All chemicals used were of the highest grade available and were, unless otherwise stated, purchased from Sigma/Aldrich, Gillingham, Dorset, UK. GW501516 was synthesised by AF ChemPharm Ltd., Sheffield, UK.

### 4.2. Transgenic Mouse Generation

Throughout this study all animals were treated in accordance with regulations contained in the Animals and Scientific Procedures Act (1996) of the United Kingdom, and with the approval of the University of Dundee ethical committee. Mice were housed in an environment with a temperature range of 19–23°C, under 12 hour light/dark cycles and given free access to food and water. The animals were fed with RM1 laboratory animal feed (SDS Ltd., Wickam, UK).

The generation of mice expressing hPPAR*δ* is described elsewhere [32]. To generate mice conditionally over-expressing a derivative of hPPAR*δ* lacking the eleven carboxy-terminal aminoacids residues (hPPAR*δ*ΔAF2) [26, 27], the coding sequence of hPPAR*δ* was amplified using primer PRMG15 (5′- CTAGTCTAGA **ATG**GAGCAGCCACAGGAGGAAGC-3′) and PRMG16 (5′- CTAGTCTAGATTAGTGCAGCGAGGTCTCGGTTTC-3′), (*Xba*I-sites underlined, ATG start codon in bold). This PCR product was cleaved with *Xba*I and cloned into plasmid pUHD10-3 (M. Gossen, unpublished, Genbank accession number U89931) creating pMGD10. The integrity of the insert ion was confirmed by sequencing and cleaved out using *Bam*HI and ligated into pAHIR1-*β*-gal [28], digested with *Bgl*II resulting in plasmid pMGD18 (hPPAR*δ*ΔAF2). The correct orientation was confirmed by sequencing. Mice were generated as for hPPAR*δ* as described previously [32]. Four founder lines on a C57BL/6 background were generated for each transgene and analysed for induction of transgene expression and suitable lines selected and brought forward for experimental analysis.

For experimental analysis, all animals were between 8 and 12 weeks of age. See figure legends of individual experiments for numbers and sex of the animals included.

Transgene expression was induced by supplementing the feed with indole 3-carbinol (I3C) at either 0.5% or 0.25% (w/w). Activation of hPPAR*δ* andhPPARD*δ*ΔAF2 transgenes in these animals was achieved by supplementing the diet with bezafibrate (0.5% w/w) or GW501516 (0.005% or 0.0025 w/w). See figure legends of individual experiments for exact dietary supplementation and time frames.

### 4.3. Determination of Transgene Detection

All animals were analysed for the presence of the transgene by PCR. DNA was extracted from ear notches and the hPPAR*δ* or hPPAR*δ*ΔAF2 transgene was amplified using primer set PRMG159 (5′-CCAACCACCCTGTCCCAGCTTG-3′) and PRMG160 (5′-ACAAACTCTGCCCTGCTCTATG-3′) using HotStarTaq DNA polymerase and Q-solution (Qiagen).

### 4.4. RNA Isolation and Semiquantitative Real-Time PCR

Total RNA from mouse tissue was isolated using the RNeasy Mini Kit (Qiagen) from kidney, liver, spleen, lung, ovary, and testis. The RNeasy Lipid kit (Qiagen) was used to isolate total RNA from brain, adipose, and breast. Total RNA isolation from heart, muscle, large and small intestines, stomach, and skin was isolated using RNeasy Mini Fibrous kit (Qiagen). All tissue samples were snap frozen in liquid N_2_ upon harvest and kept frozen until homogenization in the relevant RNA extraction kit buffers using a rotor-stator homogenizer. RNA isolation kits were used as per manufacturer's instructions, and in all cases an on-column DNAse digestion step was included prior to total RNA elution to ensure complete removal of any genomic DNA from the preparation. Purified total RNA was stored at −80°C. First strand cDNA synthesis form 200 ng of RNA was performed using Omniscript Reverse Transcription kit (Qiagen). Upon completion, the reaction was diluted in water, and a volume representing 5 ng of starting material was used for PCR reactions. The measurement of mRNA levels was achieved using quantitative real-time PCR using TaqMan chemistry on an Applied Biosystems 7900 sequence detector instrument. The primer and probe sets used to amplify hPPAR*δ* were published previously [43]. The primer and probe set used to amplify mouse *Acox1*mRNA was primer: 5′-TGACCGTTAAGGTCTTTGCAGA-3′ and 5′-AGGTTCCTCAGCACGGCTT-3′ with probe 5′-[Fam]-AACTCCCCAAGATTCAAGACAGAGCCGT-[Tamra]-3′. Mouse *Adrp *mRNA was amplified using primer 5′-CAGCCAACGTCCGAGATTG-3′ and 5-CACATCCTTCGCCCCAGT-3′; probe 5′[FAM]-TGCCAGTGCCAGAGGTGCCGT-[Tamra]-3′.

### 4.5. Lipid Measurements

Total hepatic lipids were extracted according to Folch method [44]. Lipid analysis of liver extracts was performed using RX Daytona clinical analyser (Randox, UK) following manufacturer's instructions.

### 4.6. Western Blot

Soluble lysates were prepared from frozen liver as described previously [45]. Protein concentrations were determined by the Bradford dye-binding assay (BioRad). For immunoblotting, 30 *μ*g aliquots of liver lysate were resolved by SDS/polyacrylamide-gel electophoresis, transferred to immobilon-P membranes and blocked with reconstituted dried milk (10% w/v) in TBS-tween. Membranes were probed with antihuman PPAR*δ*/NR1C2 monoclonal antibody (R&D systems) at a concentration of 1 *μ*g/mL in TBS-tween supplemented with dried milk powder (10% (w/v)). Following washing the membrane was incubated with secondary antimouse IgG coupled to horseradish peroxidase (1 : 10,000 dilution) (Dako). Bands were visualized using enhanced chemiluminescence (Millipore), and images were captured using the Fujifilm LAS3000 mini imager. To confirm equal loading of samples, blots were reprobed with antibodies against GAPDH (Sigma).

### 4.7. Statistical Analysis

Data was analysed using GraphPad Prism Software (Graphpad Software Inc., CA, USA). Statistical significance was calculated using the non-parametric Mann-Whitney *U* test. Statistical significance is described in the text and is indicated in the figures as (*P ≤ 0.05; **P ≤ 0.01; ***P ≤ 0.001).

## Figures and Tables

**Table 1:** Effect of GW501516 treatment on body weight (g) and hepatic triglycerides (mg/g of liver) in nontransgenic, hPPAR*δ* and hPPAR*δ*ΔAF2 mice.

Genotype	Treatment	Number of mice	Starting weight (g)^a^	Ultimate weight (g)^a^	*P* value^b^	Hepatic trigs (mg/g)	*P* value
Non-Tg	Control	5	24.35 ± 0.44	24.03 ± 0.53	0.1592	9.68 ± 1.18	
hPPAR*δ*	Control	5	22.27 ± 0.98	22.15 ± 1.27	0.972	15.73 ± 3.62	ns 0.152^c^
hPAR*δ*ΔAF2	Control	5	22.02 ± 1.03	21.96 ± 1.18	0.8675	52.98 ± 6.06	***0.0001^c^
Non-Tg	GW	5	21.36 ± 0.44	20.77 ± 0.53	0.1962	22.66 ± 2.31	**0.0011^d^
hPPAR*δ*	GW	5	21.78 ± 0.98	18.4 ± 1.27	*0.0179	47.53 ± 6.24	**0.0024^d^
hPAR*δ*ΔAF2	GW	5	21.97 ± 1.03	22.14 ± 1.18	0.5767	25.66 ± 2.16	**0.0023^d^

Values are the means ± S.E.M.

^a^Values adjusted for sex.

^b^ *P* value of weight difference (ultimate body weight minus initial body weight; paired *t*-test).

^c^ *P* value of difference between level of hepatic triglycerides: non-Tg control versus transgenics control (unpaired *t*-test).

^d^ *P* value of difference between level of hepatic triglycerides: control versus treated groups (unpaired *t*-test).
